# Data on the descriptive overview and the quality assessment details of 12 qualitative research papers

**DOI:** 10.1016/j.dib.2016.07.009

**Published:** 2016-07-14

**Authors:** Maia Barnabishvili, Timo Ulrichs, Ruth Waldherr

**Affiliations:** Berlin School of Public Health, Charité Universitätsmedizin Berlin, Seestr. 73, 13347 Berlin, Germany

**Keywords:** Acceptability of Health Care, Access to Health Care, Drug-resistant tuberculosis, High M/XDR-TB burden countries, Quality assessment of qualitative studies, Critical appraisal of qualitative studies

## Abstract

This data article presents the supplementary material for the review paper “Role of acceptability barriers in delayed diagnosis of Tuberculosis: Literature review from high burden countries” (Barnabishvili et al., in press) [1]. General overview of 12 qualitative papers, including the details about authors, years of publication, data source locations, study objectives, overview of methods, study population characteristics, as well as the details of intervention and the outcome parameters of the papers are summarized in the first two tables included to the article. Quality assessment process of the methodological strength of 12 papers and the results of the critical appraisal are further described and summarized in the second part of the article.

Specifications Table

**Table:** 

Subject area	Healthcare; Public Health.
More specific subject area	Drug-resistant tuberculosis; Delayed diagnosis of TB; Access to TB services; Acceptability of healthcare.
Type of data	Tables, figures
How data was acquired	Review and analysis of the relevant literature
Data format	Summarized, analyzed
Experimental factors	12 articles overviewed and analyzed here were obtained through an extensive literature review process where Titles and abstracts of 4046 initial records obtained through relevant online and offline sources, and 1796 references were screened against preliminarily developed and post-hoc inclusion/exclusion criteria.
Experimental features	12 articles, identified as relevant through the above described Search and screening process were analyzed by extracting the Standard aspects of charting process from both, scoping and systematic approaches, such as author, year of publication, study location, aims of the study, overview of methods, study population, intervention type, outcomes measures and results
Data source location	South Africa, Vietnam, DR Congo, Bangladesh, India, Malawi, China, Ethiopia, Russian Federation, Philippines
Data accessibility	All of the data are within this article.

## 1 Value of the data

• The data mostly serves to help reader to understand the review article [1] about the Acceptability barriers and their link to the TB diagnostic delay;
• The data provided here, in combination with [1], sets an example of how quality assessment of the included papers can be conducted for a scoping review article and how it can enhance the quality and value of a review. Here we hope for setting a benchmark of higher quality scoping review articles in our field and beyond;
• Two distinct methods of quality assessment of individual papers’ methodological strength were employed to produce the data presented here. Acknowledging that there is a range of alternative methodological approaches that may be employed for the same purposes, we only hope to serve as a springboard and encourage scholars to go beyond the methods used here, while also offering the thorough overview of two methods and the relevant results achieved using these methods as a reference.

## 2 Data

The data in this article consists of tables and figures providing the general overview of 12 qualitative research papers [2], [3], [4], [5], [6], [7], [8], [9], [10], [11], [12], [13] reviewed in frames of a scoping review article [1], as well as the results of methodological evaluation of the same papers.

The overview of the papers is based on the data charting/extraction process where particular aspects of each paper were extracted and analyzed systematically. Quality assessment is conducted using two different methodologies that are thoroughly described below.

## 3 Experimental design, materials and methods

To prepare a broad comparative overview of 12 included studies that is documented in Tables 1 and 2, we firstly extracted the relevant information from the individual papers through the charting process. Process of charting the data in scoping reviews is a counterpart of ‘data extraction’, which is known to be an essential part of systematic review conducting process [14].

Standard aspects of charting, described by different authors, were adopted as a framework for this process [14], [15]. The list of extracted aspects included:

1. *general information about the paper*: [*author, year of publication, type of publication, study language, study location*];
2. *study characteristics*: [*aims/objectives of the study, study design and overview of methods*];
3. *study population characteristics*: [*number of participants, disease characteristics, demographics, socio-economic status, education, occupation*];
4. *intervention/Exposure*: [*details of the described intervention and co-intervention(s)*];
5. *outcomes/measures*: [*details and the measures employed to assess primary and secondary outcomes*];
6. *results*: [*Results of study analysis*].

General information, study characteristics, and the details of study populations extracted from the 12 analyzed papers are summarized in Table 1. Papers are sorted according to their publication years. In characteristics of study participants we emphasize whether they were TB patients (former or current), people at risk, or health service providers (i.e. physicians, nurses, community health workers, or traditional healers), also whether they were interviewed individually or participated in Focus Groups Discussions (FDG).

Demographics and SES details are reported using the same scales and terminology as in the original papers.

Details of intervention and outcome elements of each of 12 papers are summarized in Table 2. Two parts of the table classify the analyzed papers according to the data source locations and publication years, accordingly. Papers are then distributed according to (a) whether they address the health services acceptability barriers (intervention of interest in the review article [1]) as a primary- or co-intervention, and (b) whether they report Delays in TB diagnosis (outcome of interest in the review article [1]) as primary or secondary outcome.

Next part of this data article deals with the methodological quality assessment of 12 papers, that employs careful and systematic examination of research to judge “its trustworthiness, and its value and relevance in a particular context” [16].

Tools for assessing the quality differ according to the study designs [17]. Since all of the 12 papers to be evaluated were qualitative research papers, *Critical Appraisal Skills Programme (CASP)* for qualitative studies [18] was selected as a primary approach. This was considered to be the most appropriate checklist as this tool:

(1) is widely used and accepted for quality assessment [19];
(2) consists of ten questions and covers the most important aspects of critical appraisal (reliability, validity, objectivity);
(3) is described to be suitable for different types of qualitative designs [19];
(4) clearly defines what is meant by each individual criterion listed and is, thus, recommended as particularly useful for scholars with little experience in qualitative research [19].

Ten questions of CASP checklist (See: Table 3) are designed in order to provide clear picture of the methodological limitations of the appraised studies^4^. Questions were answered with YES (1), when the text of the evaluated article covered the questions explicitly and provided direct answer without the need for interpretation. Answer was NO (0), when there was no information in the text that supported a positive answer, and we answered CAN’T TELL (-) when the information about an issue was provided, but it seemed to be insufficient or not specific enough for a definite answer. Detailed results of quality assessment process using CASP checklist is reported in Table 3.

**Fig. 1:**
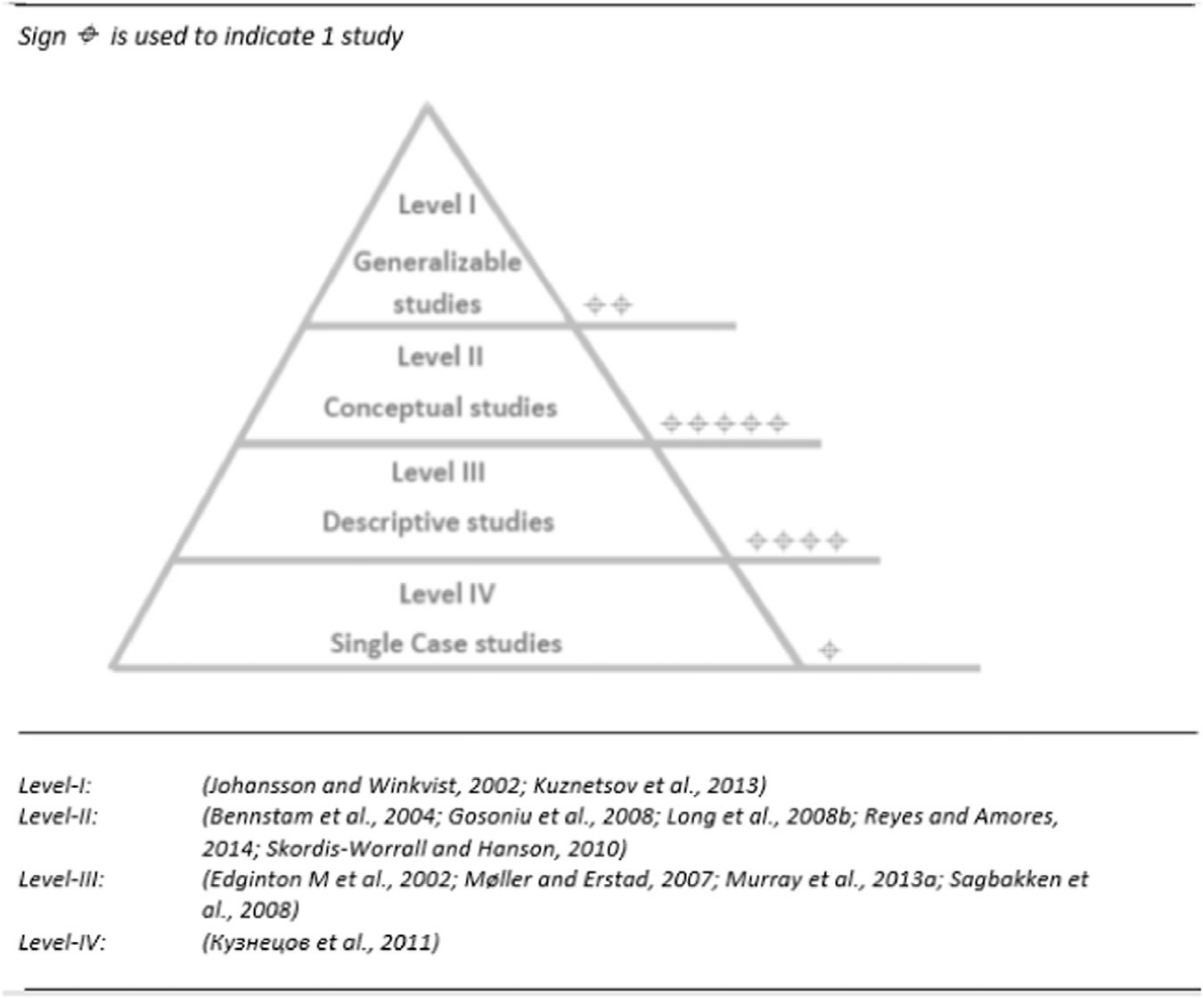
Distribution of the reviewed papers, along to the levels of “hierarchy of evidence-for-practice in qualitative research” [20].

The next step in the evaluation of the methodological clarity and/or limitations of 12 papers was to map the studies on to the hierarchical model of Daly et al. [20]. This model is suggesting the “hierarchy of evidence-for-practice in qualitative research” where four different levels of evidence are outlined. The results are reported in Fig. 1 followed by the thorough description of the process.

At the lowest level of the Fig. 1 above are the single case studies that provide information about experiences and attitudes of interviewee in a comprehensive way; besides, efforts are made to assess the applicability of findings to the region, where the interviewee come from, however applicability of the findings to other contexts as well as the saturation of data is questionable. One out of 12 papers were categorized as Level IV evidence.

Descriptive studies constitute the Level III. These are described as studies that report findings based on sample from a specific setting(s) and describe experiences, views, attitudes and actions of the interviewees in their findings without any attempts to draw any explanatory theories or creating such frameworks based on the research findings. Risk of these types of studies is that samples may often be self-selected [20]. Four studies were evaluated as Level III among 12 reviewed papers all of them being in risk for selection bias (see: Table 3). However, the studies do not have ambitious aims, about generalizing the findings and the authors did carefully indicate that their findings were based on specific settings and/or groups of people.

Higher than the level III descriptive studies, conceptual studies are positioned on the Level II in the hierarchy. This category is described as guided by a conceptual framework in their sample selection process, whereas sample often includes “a range of conceptual categories identified as significant in earlier research” [20]. We assigned 5 out of 12 reviewed papers to this level of the evidence. Gender was the main issue, guiding the sample selection in 2 out of 5 studies [11], [13]. Social and cultural status (religion, marital status, occupation) [11], as well as migration background [8], and income/poverty [5] were the other factors, playing a role in selection process. One study was based on the conceptual framework of interactions between patient and provider, whereas ethnicity and gender was considered as important aspects for selection of study participants [3]. All of the studies, assigned to this level, included comprehensive analyses of the literature around the concepts they were based on, and attempted to recognize the diversity in views of the selected groups of participants.

Finally, the generalizable studies are positioned at the top of the hierarchy. These are papers with relatively high methodological quality, which is the main prerequisite for the generalizability of the findings. They frequently provide schematic models, explaining the relation between *interventions* and *outcomes* of their research questions and are not limited to the study population only, but are applicable to a wider context. The evidence, provided by these studies may serve as indications, offering support for current practice or policy, or critique, suggesting directions for change. Two studies were identified as Generalizable studies in our work [9], [10].

## Figures and Tables

**Table 1:** Overview of the included studies:

	**Study Characteristics**		**Study population characteristics**		
	**Author (Year)/country**	**Journal/language / design**	**Description/Number**	**Demographics**	**SES**
1	Edginton et al. 2002	Published in IJTLD	303 interviewees; 186 FGD participated	‘Younger than 15’ (12%), ‘15–59’ (76%), ‘>60’ (12%)	< 4 yr of education (44%) some primary/secondary educ. (56%)
	South Africa	English	TB patients, & community members	Male (72%), female (28%)	0 yr of employment (30%); employed (70%)
		Mixed Approach: quantitative plus qualitative (FDGs);			
2	Johansson and Winkvist, 2002	Published in QUAL HEALTH RES	24 TB patients (ongoing/ recent history of TB);	age group: 17-74	
				15 male/9 female patient	adolescents to pensioners
	Vietnam				
				8 males/7 female provider	
		English	15 health care providers;		
		Qualitative approach (In-depth Interviews)			
3	Bennstam et al., 2004	Published in QUAL HEALTH RES	49 participants with and without TB;	age group: 21-44	–
	DR Congo			26 males / 23 females	–
		English			
		Qualitative approach (FGDs /grounded theory)			
4	Møller and Erstad, 2007	Published in Int J Equity Health English	59 participants:	age group: 15–79	0 yr of schooling (10%);
			Community H-workers. (8), TB patients (8), High school pupils (7), Out-of-school youth (8), adult women (8), Adults (Mixed) (7), Older adults (8), Traditional healers (5)	22 males /37 females	
	South Africa				
		Qualitative approach (FGDs)			Primary or high education (66%);
					Students (25%), unemployed (25%), community health workers (25%) social pensioners, (25%)
5	Gosoniu et al., 2008	Published in IJTLD	329 participants	Age group: ----	–
			102 (Bangladesh),	158 males/50 females	Nil (11.6%), Student (1.6%), Housewife (20.7%), Unskilled labor (10.5%), Skilled labor (16.7%), Trade/business (17.5%), Farmer (7.5%), Other (13.9%)
	Bangladesh, India, Malawi;	English	127 (India),		
				66 males/61 females	
		Qualitative research (Semi-structured interviews)	100 (Malawi)		
				50 males/50 females	
6	Long et al., 2008	Published in: *BMC Health Services Research*	1005 participants (776 resid. /229 migr.)	Age group: >15	Elementary school or less =44% of migrants/37% of residents
		English			
		Mixed Approach: quantitative plus qualitative (in-depth/FGD)			
				Both males and females (no further specification)	

					Lowest income group:
					63% migrants/47% residents
	China				
			60 individual interviews (20 TB suspects, 17 TB patients, 23 health workers)		
			12 FGD groups		
7	Sagbakken et al., 2008	Published in: QUAL HEALTH RES	10 TB patients on treatment, 11 with interrupted treatment	Age group: 18-67	0 yr of education – 6 participants
	Ethiopia			11 males / 10 females	1–6 yr of education– 6 participants
			5 health professionals;		
					7–13 yr of education – 12 participants
		English			
		Qualitative approach (In-depth Interviews/FGDs)			
8	Skordis-Worrall and Hanson, 2010	Published in: IJTLD	n≈56	Age group: 20–39	–
	South Africa	English	8 focus groups, each with 6 to 8 part., stratified by gender, ethnicity, TB status	4 FG of males/4 of females	–
		Qualitative approach (FGDs)			
9	Кузнецов et al., 2011	Published in: *Экология человека*	n=1	Age: 38	–
		Russian			
		Qualitative approach (In-depth Interview)			
			(patient with active TB, with history of imprisonment)	male	Physical worker
	Russian Federation				
10	Kuznetsov et al., 2013	Published in: BMC Public Health	23 participants in 5 FGD with 5–6 informants in each;	Age group: 27–53 years	0 yr of schooling – 2 participants
	Russian Federation			9 females/14 males	9 yr of schooling - 12 participants
			New cases with a drug-susceptible form of Tuberculosis.		
					College graduation - 9 participants
		English			
		Qualitative approach (FGDs /grounded theory)			–
11	Murray et al., 2013a	Published in: Health policy & plan.	(Communities of eight South African township sites of Cape Town, with high burden of undiagnosed TB/HIV	–	–
	South Africa			–	–
		English			
		Retrospective use of qualitative data (Fieldwork Data)			
12	Reyes and Amores, 2014	Discussion Paper from Philippine Institute for Developm. Studies	21 participants in three FGD	Age group: 17–64	–
					–
	Philippines			11 males/10 females	
		English			
		Qualitative approach (FGDs)			

**Table 2:** Overview of the included studies (Intervention/Outcome elements of PICO).

*Studies are classified according to whether they include the Intervention and Outcome of interest as primary or secondary targets, whereas these data is presented according to the countries of origin, as well as the years of publication of the studies.*					
	**SUM**	**Acceptability barriers discussed as:**		**Delays in TB diagnosis discussed as:**	
		intervention	Co-intervention	Primary Outcome	Secondary Outcome
Countries:	**12**	**9**	**3**	**7**	**5**
Bangladesh	1^a^	1	–	1	–
China	1	1	–	1	–
DR Congo	1	–	1	–	1
Ethiopia	1	1	–	–	1
India	1^a^	1	–	1	–
Philippines	1	1	–	1	–
Russian Fed.	2	2	–	2	–
South Africa	4	2	2	2	2
Vietnam	1	1	–	–	1
Years of Publication:	**12**	**9**	**3**	**7**	**5**
2002	2	2	–	1	1
2004	1	–	1	–	1
2007	1	–	1	–	1
2008	3	3	–	2	1
2010	1	1	–	1	–
2011	1	1	–	1	–
2013	2	1	1	1	1
2014	1	1	–	1	–

a One Multi-country study, reporting data from both India and Bangladesh, is presented here separately, for both countries.

**Table 3:** critical appraisal of methodological quality of included studies according to the CASP checklist.

**Checklist question**Yes(1), No(0), and can’t tell(-) are the possible answers		**Studies**^a^											
		**1**	**2**	**3**	**4**	**5**	**6**	**7**	**8**	**9**	**10**	**11**	**12**
**1**	**Was there a clear statement of the aims of the research?***‘Consider: goals of the research, why it is important and its relevance (this should be explicitly stated in the abstract or introduction)’*	1	1	1	1	1	1	1	1	1	1	1	1
**2**	**Is a qualitative methodology appropriate?***‘Consider if the research seeks to interpret or illuminate the actions and/or subjective experiences of research participants’*	1	1	1	1	1	1	1	1	1	1	1	1
**3**	**Was the research design appropriate to address the aims of the research?***‘Consider: if the researcher has justified the research design (e.g. have they discussed how they decided which methods to use?). We will answer “YES” only in the case we can find in the text the justification of the research design.’*	0	0	1	0	1	0	–	0	1	1	0	0
**4**	**Was the recruitment strategy appropriate to the aims of the research?***“YES” only in the case the researchers provide information enough to conclude that there is no selection bias. In case you identify a selection bias OR authors don´t provide information about the recruitment strategy - answer “NO”’*	–	1	1	–	–	1	0	1	1	1	0	1
**5**	**Were the data collected in a way that addressed the research issue?***‘YES” in: (1) the researcher discussed saturation of data AND (2) the researcher made the methods explicit (e.g. how interviews were conducted) AND (3) the form of data is clear (e.g. tape recordings, video material, notes etc)’.*	0	0	1	0	0	1	1	1	1	1	0	0
**6**	**Has the relationship between researcher and participants been adequately considered?***‘Consider: whether researcher critically examined own role, potential bias and influence. If information is reported either in the methodology section (how they avoided this bias) or in the limitations (acknowledging the bias) - answer “YES”. Otherwise - “NO’”.*	1	1	1	1	0	–	0	1	0	1	1	0
**7**	**Have ethical issues been taken into consideration?***‘Consider: if approval has been sought from the ethics committee’*	1	1	1	1	0	1	1	1	0	1	1	0
**8**	**Was the data analysis sufficiently rigorous?***(1) Sufficient data are presented to support the findings (i.e., quotes included) AND (2) type of analysis used is reported (e.g. thematic analysis, grounded theory…) AND (3) There is an agreement between primary data and secondary data (the results of the authors has to correspond with the information they extracted). (4) Report of triangulation (more than one analyst)’*	1	1	1	1	1	1	1	1	1	1	1	1
**9**	**Is there a clear statement of findings?***“YES” if: (1) Summary of the results is presented in the discussion. (2) evidence is discussed adequately’*	1	1	1	1	1	1	1	1	1	1	1	1
**10**	**How valuable is the research?***‘Answer “YES” only if implications of the paper for research OR for practice OR for policy are reported’*	1	1	0	0	1	1	1	0	0	1	1	1
**∑**		**7**	**8**	**9**	**6**	**5**	**8**	**7**	**8**	**7**	**10**	**7**	**6**

a Study numbering should be interpreted as follows: 1. [12], 2. [10], 3. [13], 4. [7], 5. [11], 6. [8], 7. [4], 8. [3], 9. [2], 10. [9], 11. [6], 12. [5].

## Footnotes

4 Full version of the check list can be accessed online: http://media.wix.com/ugd/dded87_29c5b002d99342f788c6ac670e49f274.pdf

## References

[1] M. Barnabishvili, T. Ulrichs, R. Waldherr, Role of acceptability barriers in delayed diagnosis of Tuberculosis: Literature review from high burden countries. Acta Trop. 2016 Sep;161:106–113. 10.1016/j.actatropica.2016.06.014 27311390

[2] В.Н. Кузнецов, et al., *«я не бегаю по докторам, пока могу двигаться»: нарративный анализ интервью с пациентом, имеющим опыт поздней диагностики туберкулеза; “i never go to doctors, as long as i can move”: a narrative analysis of interview with a patient having tuberculosis diagnostic delay experience.* Экология человека, 2011. 11: p. 54-58.

[3] Skordis-Worrall J., Hanson K. (2010). Confusion, caring and tuberculosis diagnostic delay in Cape Town, South Africa. J. Tuberc..

[4] Sagbakken M., Frich J.C., Bjune G.A. (2008). Perception and management of tuberculosis symptoms in Addis Ababa, Ethiopia. Qual. Health Res..

[5] K. Reyes, J. Amores, Barriers of Early TB Diagnosis among the Poor in Highly Urbanized Areas in the Philippines, 2014: dirp4.pids.gov.ph.

[6] E. Murray, et al., High levels of vulnerability and anticipated stigma reduce the impetus for tuberculosis diagnosis in Cape Town, South Africa*,* 2013, 28(8610614, f9q): pp. 410–418. 10.1093/heapol/czs072 22945548

[7] Møller V., Erstad I. (2007). Stigma associated with tuberculosis in a time of HIV/ AIDS: narratives from the Eastern Cape, South Africa. South Afr. Rev. Soc..

[8] Long Q. (2008). Barriers to accessing TB diagnosis for rural-to-urban migrants with chronic cough in Chongqing, China: a mixed methods study. BMC Health Serv. Res..

[9] Kuznetsov V. (2013). Hopelessness as a basis for tuberculosis diagnostic delay in the Arkhangelsk region: a grounded theory study. BMC Public Health.

[10] Johansson E., Winkvist A. (2002). Trust and transparency in human encounters in tuberculosis control: lessons learned from Vietnam. Qual. Health Res..

[11] Gosoniu G.D. (2008). Gender and socio-cultural determinants of delay to diagnosis of TB in Bangladesh, India and Malawi. Int. J. Tuberc. Lung Dis..

[12] Edginton M E., Sekatane C S., Goldstein S J. (2002). PatientsÕ beliefs: do they affect tuberculosis control? A study in a rural district of South Africa. Int. J. Tuberc. Lung Dis..

[13] Bennstam A.L., Strandmark M., Diwan V.K. (2004). Perception of tuberculosis in the Democratic Republic of Congo: wali ya nkumu in the Mai Ndombe district. Qual. Health Res..

[14] Arksey H., O׳Malley L. (2005). Scoping studies: towards a methodological framework. Int. J. Soc. Res. Methodol..

[15] Armstrong R. (2011). Cochrane Update. ׳Scoping the scope׳ of a cochrane review. J. Public Health.

[16] Burls A. (2009). What is critical appraisal?. Evid. Based Med..

[17] Centre for Reviews and Dissemination (2008). Systematic Reviews, CRD’s guidance for undertaking reviews in health care.

[18] Critical Appraisal Skills Programme, C. (2013). Making sense of evidence. 10 questions to help you make sense of qualitative research..

[19] K. Hannes, Chapter 4: Critical appraisal of qualitative research, in Supplementary Guidance for Inclusion of Qualitative Research in Cochrane Systematic Reviews of Interventions, B.A. Noyes J, Hannes K, Harden A, Harris J, Lewin S, Lockwood C, Editor, Cochrane Collaboration Qualitative Methods Group, 2011.

[20] Daly J. (2007). A hierarchy of evidence for assessing qualitative health research. J. Clin. Epidemiol..

